# Multi-Scale Computational Enzymology: Enhancing Our Understanding of Enzymatic Catalysis

**DOI:** 10.3390/ijms15010401

**Published:** 2013-12-31

**Authors:** Rami Gherib, Hisham M. Dokainish, James W. Gauld

**Affiliations:** Department of Chemistry and Biochemistry, University of Windsor, Windsor, ON N9B 3P4, Canada; E-Mails: gherib@uwindsor.ca (R.G.); dokaini@uwindsor.ca (H.M.D.)

**Keywords:** enzyme catalysis, density functional theory (DFT) cluster method, quantum mechanics/molecular mechanics (QM/MM), molecular dynamics (MD) simulations, molecular docking

## Abstract

Elucidating the origin of enzymatic catalysis stands as one the great challenges of contemporary biochemistry and biophysics. The recent emergence of computational enzymology has enhanced our atomistic-level description of biocatalysis as well the kinetic and thermodynamic properties of their mechanisms. There exists a diversity of computational methods allowing the investigation of specific enzymatic properties. Small or large density functional theory models allow the comparison of a plethora of mechanistic reactive species and divergent catalytic pathways. Molecular docking can model different substrate conformations embedded within enzyme active sites and determine those with optimal binding affinities. Molecular dynamics simulations provide insights into the dynamics and roles of active site components as well as the interactions between substrate and enzymes. Hybrid quantum mechanical/molecular mechanical (QM/MM) can model reactions in active sites while considering steric and electrostatic contributions provided by the surrounding environment. Using previous studies done within our group, on OvoA, EgtB, ThrRS, LuxS and MsrA enzymatic systems, we will review how these methods can be used either independently or cooperatively to get insights into enzymatic catalysis.

## 1. Introduction

Enzymes, being essential to life, have long been the focus of considerable efforts, in order to understand their properties and biochemical roles. In addition to the fundamental chemical insights to be gained, it may also lead to the development of new therapeutic drugs and synthetic catalytic systems [1–3]. Central to a complete understanding of an enzyme is the elucidation of its catalytic mechanism. Numerous investigations have ascribed the origin of enzymes rate-enhancing power to several possible factors including steric effects and transition-state stabilization [4]. Unfortunately, the precise driving forces and atomistic-level details underlying many mechanisms remain unknown or are the subject of considerable discussion and, at times, controversy [5–7]. Various “models” have been developed to explain their machinery: some have focused on the possible role of the overall structure and corresponding conformational variations [8–10], while others have focused on the role of a few chemical species and functional groups within their active sites [11–13]. Collectively this has led to the gradual development of a more holistic picture of enzymatic catalysis. That is, enzymes are increasingly seen as systems that must be considered both at atomistic and macroscopic levels.

### 1.1. Experimental and Computational Enzymology

Experimental biochemists routinely use a wide variety of techniques and methods to study the structure, reactivity and dynamics of enzymes and their corresponding biochemical processes. Indeed, methods such as X-ray crystallography, mutagenesis, kinetic isotope studies, mass spectrometry, and ultraviolet-visible, fluorescence, infrared, circular dichroism and nuclear magnetic resonance spectroscopies can each provide insights into the system under study.

Key aspects of enzymatic processes can be acquired through the complementary application of experimental and theoretical methodologies. Indeed, the shortcomings of one can often serves as the strengths of the other. For example, as one of the limitations of experimental methods is their inability to directly characterize transition states and reactive intermediates constitutes, one can often rely on computational methods to model the different species along the reaction pathway. Furthermore, experimental methods, which provide extensive mechanistic insights, often constitute the pillars of computational studies in addition to serving as benchmark for the validation and development of computational methods.

Due to the complexity of enzymatic systems, many computational models are derived from X-ray crystal structures. As the latter represents a powerful tool that provides detailed insights into the 3D structure of an enzymatic system, the resulting structures are often successfully used to infer reaction steps or entire mechanisms. In conjunction with crystal structures, computational methods can model the dynamic properties of enzyme and their ability to undergo conformational changes. Furthermore, as crystal structures do not always correspond to enzymes’ endogenous state, computational methods can be used to remedy the effect of crystallization by solvating the enzyme, and through simulated annealing, remove lattice-packing effects. Since these effects may result in misleading inferences [14–18], the synergetic use of crystallography and computational studies presents itself as a valuable tool in enzymology.

### 1.2. Computational Enzymology Methods

Recent years have seen the emergence of computational chemistry methods in the fields of enzymology. This has been facilitated by the development of methods that collectively span a broad range of computational approximations and chemical models such as: (i) docking; (ii) molecular dynamics (MD) simulations; (iii) quantum mechanical-chemical cluster (QM-cluster); and (iv) hybrid quantum mechanics/molecular mechanics (QM/MM) methods. Consequently, whether these methods have been used to study enzymes at the quantum mechanical level (while investigating orbital steering and tunneling effects) [19,20] at an intermediate level (while looking at the different substrate conformations inside active sites) [17] or at the macroscopic scale (where domain motions and conformational transitions could potentially play rate-limiting roles in enzyme catalysis) [21], they have become indispensible tools in the study of biomolecules. Many excellent books and reviews have previously described some of the computational methods that are commonly employed in multi-scale computational investigations, and listed above. Hence, only a brief overview is provided herein with an emphasis on their possible application to enzymology.

As noted above, X-ray crystallographic structures do not always possess the “natural” substrate(s) bound to the enzyme’s binding site(s). In some cases, receptor bound inhibitors or substrate-analogues were used to investigate substrate-receptor interactions. In other cases, the complete absence of substrate or substrate-analogues in available crystal structures prohibits the deduction of these interactions. In such instances, molecular docking methods can be used to model sets of probable “natural” substrate binding modes with scoring algorithms providing some measure of the preference of the resulting complexes [22].

Molecular dynamics (MD) simulations enable one to model the time-dependent trajectories of atoms within a system. Such methods require atomic accelerations to be computed and combined with the positions and velocities at a given time *t* to calculate new positions and velocities at time *t* + δ*t*. During each time-step, forces are assumed to be constant but must be recalculated for each time step. Molecular mechanics (MM) forcefields are typically used to describe the atoms or groups within the chemical system. Although such methods cannot be used to investigate “electronic” processes such as bond making and breaking, they enable sampling of the configuration space thereby allowing one to explore and consider multiple points on the potential energy surface. Furthermore, very extensive chemical systems such as solvated enzyme···substrate complexes can be modeled in their entirety. In addition, one can incorporate pressure, temperature and volume effects in a time dependent manner. Thus, for example, one can use such methods to obtain average structures of solvated, thermally relaxed (at 298 K) enzyme-substrate complexes [23].

In a quantum mechanical-cluster approach [24] a small to modest sized (e.g., ≤200 atoms) chemical model of the complete enzymatic system is used. Typically, such a model is derived from crystal structures, although this is not an absolute requirement (see below). The model generally includes the substrate, its reactive component and key active site residues that are either mechanistically important or interact with the substrate. In the case of metalloenzymes, where a substantial part of the catalysis is often determined by the electronic properties of the metal ion, a valid model should contain the metal as well as its immediate coordination environment [25–27]. A select few atom centers, at a remote region from the reactive region, are held fixed in order to maintain the integrity of the chemical model. The cluster can be further complemented by modeling the rest of the surrounding enzyme via a polarizable continuum (PCM) solvation method. With regards to the choice of QM method, due to their computational speed and ability to provide reliable results, the vast majority of QM-cluster studies use density functional theory (DFT) methods (e.g., B3LYP) [28]. The resulting approach can model reactants, intermediates, transition states and product complexes, providing insights into their electronic, kinetic and thermodynamic attributes.

Hybrid QM/MM methods can be applied to extensively large chemical models as they partition them into several sub-systems (layers), which can then be treated at different levels of theory. Typically, the model is divided into two layers with the high-layer containing the enzyme’s reactive region and the second, low-layer containing the surrounding protein and environment. The QM/MM formalism can in principle utilize a diverse combination of methods. In practice, however, the high-layer is commonly described using a DFT method (although it should be stated that semi-empirical methods have been extensively used especially in QM/MM-MD) while the low-layer is described by an MM forcefield such as AMBER [29–32], CHARMM [33–35], GROMOS [36–38] or OPLS-AA [39–41]. The latter methods have been parameterized specifically for biochemical systems such as proteins and nucleic acid complexes. The energy of the total system can be partitioned in two ways through either an additive (QMMM) [42–44] or subtractive (ONIOM) [45] scheme as seen in Equations (1) and (2), respectively.

(1) $$E_{\text{Tot}}^{\text{QM}/\text{MM}}=E_{\text{RE}}^{\text{QM}}+E_{\text{SE}}^{\text{MM}}+E_{\text{RE}\cap \text{SE}}^{\text{QM}\cap \text{MM}}$$

(2) $$E_{\text{Tot}}^{\text{ONIOM}}=E_{\text{Tot}}^{\text{MM}}-E_{\text{RE}}^{\text{MM}}+E_{\text{RE}}^{\text{QM}}$$

The additive scheme requires a QM calculation on the reactive environment ( $E_{\text{RE}}^{\text{QM}}$), an MM calculation on the surrounding environment ( $E_{\text{SE}}^{\text{MM}}$) followed by an energy coupling representing the interaction between the QM and MM layers ( $E_{\text{RE}\cap \text{SE}}^{\text{QM}\cap \text{MM}}$). In contrast, the subtractive scheme as used in ONIOM requires an MM calculation on the entire system ( $E_{\text{Tot}}^{\text{MM}}$) and both an MM ( $E_{\text{RE}}^{\text{MM}}$)and QM ( $E_{\text{RE}}^{\text{QM}}$)calculation on the reactive environment. Thus, QM/MM methods couple the speed of MM methods with the ability of QM methods to model chemical reactions. Furthermore, they enable one to better model the non-homogeneous environment around the active site.

While powerful, each computational method suffers from inherent limitations. Although progress in computational software has allowed them to become increasingly user friendly and widespread, they should not be used in a “black-box” matter. For instance, common scoring functions often do not correlate with experimental binding affinities. Furthermore, limitations in search algorithms and scoring functions renders only 60% of docking results as reasonable [46]. Classical molecular dynamic simulations are often poorly equipped for certain systems (such as transition metals) and simulate time-scales that are often too short to appropriately model key biological processes (such as substrate binding) [47]. A QM-cluster approach can at times inaccurately model entropic and solvation effects. The former allows the calculation of the free energy surface, which unlike the potential energy surface, corresponds to macroscopic observables [48]. Furthermore, QM-clusters cannot model the inherent polarization present in enzymatic systems, which are often key to their enzymatic mechanisms. Such limitations are to be considered in addition to those related to a lack of electron correlation for each QM method. Amongst the diverse shortcomings of QM/MM methods, the misrepresentation of the QM/MM boundaries and the interactions between the two layers are noteworthy examples [49]. Certainly, experimental methods can be used to assess and enhance the performance of computational techniques and even inspire their development.

In this review, several examples of a multi-scale approach to computational enzymology are presented. This review comes at a fortunate time since shortly prior to its publication, the 2013 Nobel Prize in chemistry was awarded to Karplus, Levitt and Warshel “for the development of multi-scale models for complex chemical systems” [50]. It is also noted that an increasing number of studies can be found in the literature that also provide wonderful examples of the enhanced understanding arising from and the capabilities of employing such a computational approach. For the purposes of this review, however, examples are presented from our own work and namely will focus on the synergistic application of two or more methods within the areas of docking, MD, QM-cluster and ONIOM (QM/MM) to investigating biocatalysis.

## 2. Computational Enzymatic Studies

### 2.1. OvoA and EgtB: Characterizing Potential Mechanistic Oxidants

5-Histidylcysteine sulfoxide synthase (OvoA) and 2-histidyl-γ-glutamyl cysteine sulfoxide synthase (EgtB) activate dioxygen and react it with histidine and cysteine derivatives to synthesize sulfur-containing antioxidants. Although no X-ray crystal or NMR structures are available, some features related to their structure and activities have been experimentally deduced or determined. In particular, in both enzymes an iron is ligated to two histidyl imidazoles and a carboxylate side-chain. Because of their common chemistry and similar structural features, it has been suggested that they share similar catalytic mechanisms [51].

**Scheme 1.**
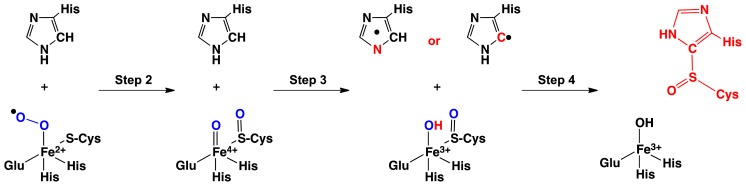
Schematic representation of the experimentally proposed mechanism for biosynthesis of a histidyl-sulfoxide derivative (adapted from [53]).

In particular, the mechanism of OvoA has been proposed to occur in four steps (Scheme 1) [52]. First, cysteine and O_2_ binds to the Fe(II) center to give an Fe(III)O_2_^·−^ containing complex. The Fe(III)O_2_^·−^ then attacks the ligated cysteine while undergoing homolytic O–O bond cleavage to form a sulfoxide···Fe(IV)=O complex. In the subsequent step, the latter complex oxidizes the co-substrate histidine’s imidazole via abstraction of a H· from either from the histidyl’s C_δ_–H, forming a C-centered radical, or N_δ_–H to give a π-delocalized radical [52]. In the fourth and final step, the imidazole radical attacks the sulfoxide, forming a histidyl-cysteinyl sulfoxide derivative. Intriguingly, it is unclear why a sulfoxide intermediate is formed but may be related to the need to form the powerful Fe(IV)=O oxidant in order to oxidize the histidine co-substrate’s imidazole R-group.

As noted above, no experimental X-ray crystal or NMR structures are available of OvoA. Hence, we chose to use of a QM-cluster based approach to investigate possible Fe-coordination environments and importantly, their impact on the redox potentials of mechanistically relevant intermediates [53]. The chemical model included the Fe ion, the R-groups of the residues to which it is ligated, as well as the three cosubstrates; O_2_, the imidazole of histidine, and cysteine. The surrounding protein environment was included via use of an integral equation formalism (IEF-PCM) [54] solvation model with a dielectric constant of 4.0. The entire model and free energies were then described at the IEF-PCM-B3LYP/6-311G(2df,p)//B3LYP/6-31G(d) + Δ*G*_cor_ level of theory, where Δ*G*_cor_ corresponds to the Gibbs free energy corrections added to electronic energies and obtained via vibrational frequency calculations. In total, due in part to the number of possible coordination models and spin-states (multiplicities), 196 possible active site model complexes were examined.

First, the oxidation of imidazole (a model of the mechanistically relevant component of the histidine co-substrate) was considered. This can potentially occur via one of three pathways: (i) an electron transfer (ET); or a proton-coupled electron transfer (PCET) from either a (ii) imidazole nitrogen (*i.e.*, N_δ_–H) or (iii) carbon (*i.e.*, C_δ_–H). Specifically, the free energy in a SHE reference state for each process was calculated via a first principle quantum statistical mechanical approach [53]. Oxidation of imidazole via an ET process corresponds to an increase in 186.0 kJ mol^−1^ in free energy and hence is non-spontaneous. Similarly, H· abstraction from the C_δ_–H or N_δ_–H moieties of an imidazole via a PCET process corresponded to an increase in 250.1 and 171.0 kJ mol^−1^ in free energy, respectively. Thus, oxidation of imidazole would appear to preferably proceed via a PCET process involving H· abstraction from the imidazole’s N_δ_–H group. Importantly, its required energy can now help provide insights into the mechanistic feasibility of a particular oxidant complex (see below).

**Scheme 2.**
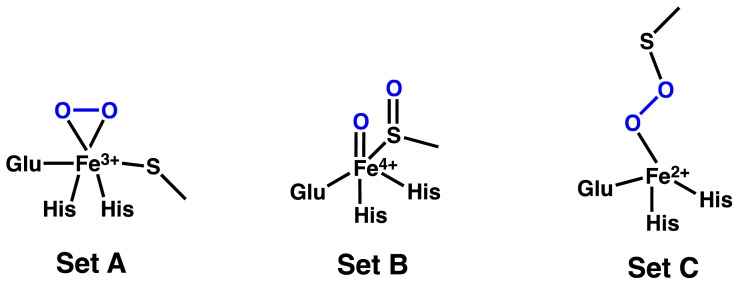
Schematic representation of the mechanistic oxidant complexes considered: those containing a (**A**) Fe(III)O_2_^·−^; (**B**) sulfoxide···Fe(IV)=O; or (**C**) Fe–OO–S_Cys_ crosslink moiety.

Experimentally, three of the ligands to the Fe centre were concluded to be the R-groups of two histidyl’s and a glutamate while a fourth ligand is oxygen or O_2_. However, it is unclear if the cysteine binds mono- or bidentately to the Fe centre. Hence, a series of active site-inspired oxidant complexes were generated, which in some cases had water as a sixth ligand. The resulting complexes, which in total numbered 196, could be classified as belonging to one of three “model sets” depending on whether they contained an: (A) Fe(III)O_2_^·−^; (B) sulfoxide···Fe(IV)=O; or (C) Fe–OO–S_Cys_ crosslink moiety (Scheme 2). The free energy of their reduction via ET and PCET was then calculated for each complex.

First, the possible spin-states of each initial complex were examined within the QM-cluster approach. For all sets of models the preferred ground state of the initial non-reduced metal system was a septet spin-state. For the reduced systems, however, those obtained via an ET process preferred a quartet and sextet ground spin-states (depending on the particular complex) while all systems obtained via a PCET process preferred a quartet ground spin-state.

For model Set A, complexes reduction via an ET process was revealed to be endergonic by at least 60.4 kJ mol^−1^ and hence, non-spontaneous. On the other hand, reduction via a PCET process was exergonic with calculated free energies ranging from −55.6 to −84.7 kJ mol^−1^. Thus, while their reduction via PCET is favorable, it is still significantly lower than the inherent energy required to oxidize a histidine’s R-group imidazole. That is, the mechanistic oxidant in OvoA is unlikely to correspond to an active site containing an Fe(III)O_2_^·−^ moiety.

The previously experimentally proposed mechanistic oxidant is an Fe(IV)=O type complex [52]. As noted above, a range of models of possible sulfoxide···Fe(IV)=O containing active site complexes (*i.e.*, Set B) were considered, as well as their reduction via an ET or PCET process. Similar to Set A, reduction of any of the Set B complexes via an ET process was calculated to be endergonic, though now by at least 44.4 kJ mol^−1^. Again, this indicates that such a reduction process is inherently unfavorable. In contrast, reduction via a PCET process was calculated to be thermodynamically very favorable with free energies ranging from −114.1 to −146.5 kJ mol^−1^. That is, active sites containing an sulfoxide···Fe(IV)=O moiety are inherently more oxidizing than the Fe(III)O_2_^·−^ containing complexes (Set A). However, as observed for the Set A species, their oxidizing potentials are still not inherently sufficient to oxidize the histidine co-substrate’s side chain imidazole.

Complexes containing an Fe–OO–S_Cys_ moiety (Set C) had not previously been considered in the proposed mechanism for OvoA and EgtB, but had been investigated computationally in cysteine dioxygenase (CDO) [55]. Unlike the complexes of Sets A and B, reduction of Set C complexes via an ET process resulted in mechanistically infeasible complexes. In contrast, their reduction via a PCET process was found to be significantly exergonic and thus favorable with free energies ranging from −203.9 to −268.3 kJ mol^−1^. In fact, Fe–OO–S_Cys_ containing complexes within Set C have sufficient oxidizing power to overcome the inherent energy required to oxidize the histidine co-substrates imidazole via PCET; *i.e.*, effectively abstracting a H· from the imidazole’s N_δ_–H or C_δ_–H moieties. However, these results do more than affirm that a Fe–OO–S_Cys_ intermediate may be the required mechanistic oxidant in the enzymes OvoA and EgtB. They also provide invaluable mechanistic insights including the possible reason for formation of a sulfoxide intermediate; it is a result of reducting of a Fe–OO–S_Cys_ intermediate.

**Scheme 3.**
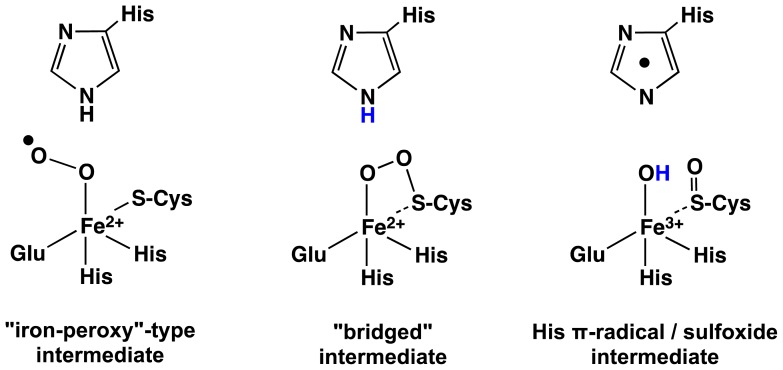
Schematic representation of computationally proposed intermediates in the formation of histidyl-sulfoxide (adapted from [53]).

From the QM-cluster study a possible mechanistic pathway for OvoA and EgtB can be suggested and is shown in Scheme 3.

As described above, even in the absence of a crystal structure a large amount of chemical and mechanistic insight can be gained via a well-chosen QM-cluster approach. In this specific case, such a method was used in conjunction with a first principles quantum statistical mechanical approach for redox properties. Significantly, while the present results do not conclusively elucidate the mechanisms of OvoA and EgtB, they provide insights into their catalytic mechanisms and active sites. In addition, however, they also give broad and applicable fundamental insights into the reduction of imidazole and importantly, the effects of a Fe centre’s coordination environment on particular Fe-complexes’ redox properties.

### 2.2. Threonyl-tRNA Synthetase (ThrRS): Identifying a Mechanistic Base?

**Scheme 4.**
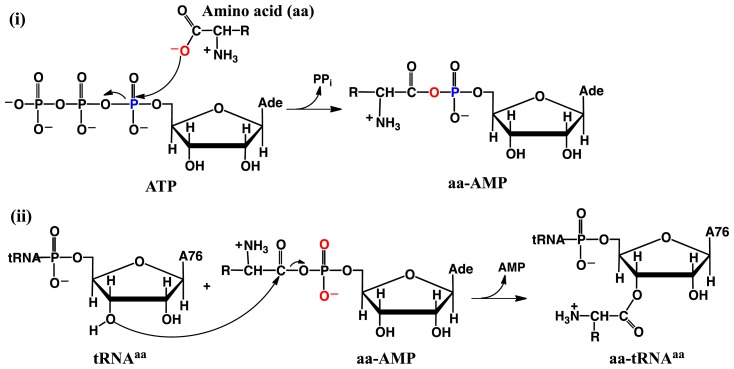
General overall mechanism of (i) amino acid activation and subsequent (ii) aminoacylation of the cognate tRNA catalyzed by aminoacyl-tRNA synthetases.

Aminoacyl-tRNA synthetases (aaRS’s) are a ubiquitous class of enzymes. They are perhaps best known for their central role in protein synthesis. Specifically, they catalyze the aminoacylation of their cognate tRNA [56–59] via the two half reactions shown in Scheme 4 [59–61]. First, the amino acid is activated by reacting with ATP to give an aminoacyladenylate (aaAMP). Then, within the same active site, they catalyze the transfer of the aminoacyl moiety of aaAMP onto the 2′- or 3′-oxygen of the Ado76 residue of the cognate tRNA (tRNA^aa^).

In the second half reaction, the ribose oxygen of Ado76 nucleophilically attacks the carbonyl carbon of the aminoacyl moiety. However, first, a suitable base must activate the oxygen. In fact, aaRS’s active sites have been observed to generally lack such residues [61–63]. Hence, it has been thought that aaRS’s may use a substrate assisted mechanism wherein one of the substrate’s own non-bridging phosphate oxygens (the pro-R/S oxygens) acts as the missing catalytic base [61]. Recently, Francklyn and coworkers examined threonyl-tRNA synthetase catalyzed aminoacylation [63]. In particular, their mutagenesis studies indicated that replacing the pro-S or -R oxygens by sulfur had little effect indicating that they were not catalytically essential [63]. On the other hand, mutation of the active site histidyl (His309) caused a more significant decrease in the rate of reaction. Hence, it was proposed that His309 directly or non-directly (via a water molecule) deprotonates the Ado76-2′-OH moiety of the cognate tRNA. The resulting 2′-O^−^ then deprotonates the adjacent Ado76-3′-OH group, thereby enabling the 3′-oxygen to better act as a nucleophile [63,64].

Recently, we have used several computational methods such as MD and QM-cluster approach in a complementary fashion to elucidate the nature of the active site base in the second half reaction of theronyl-tRNA [59,62]. Unfortunately, while several X-ray structures are available for ThrRS, none have both ThrAMP and tRNA^Thr^ bound [62]. Therefore, we began by modifying an available X-ray structure of ThrRS···(tRNA^Thr^ + AMP) (PDB code: 1QF6 [65]) to include the threonyl moiety [53]. The latter’s positioning was verified by comparing to other available X-ray crystal structures. Although His309 has initially been proposed to act as a catalytic base, for completeness and because its pKa is close to 7, we have also considered the use of a protonated His309 in which it could act as an acid. MD simulations were performed on all four complexes in order to examine their fully-bound active site structures and the consistency of the catalytically relevant interactions. Analysis of atomic trajectories indicated that in both neutral and protonated His309 (His309-H^+^), the addition of a water bridge between His309 and Ado76-2′-OH negatively impacted the substrate positioning and the consistency of key mechanistic interactions. In contrast, when no additional water was added, for both neutral and protonated His309, the tRNA^Thr^ and ThrAMP substrates were better positioned for reaction, and important active site interactions were significantly more consistent.

Although previous MD simulations provided some useful insights into the possible roles of the active site residues, it cannot be used alone to elucidate the mechanism. Subsequently, for both neutral and protonated His309 without a “bridging H_2_O”, we obtained suitable chemical models for use in a QM-cluster study on the catalytic aminoacylation mechanism of ThrRS [58]. Specifically, the chemical cluster included the active site Zn(II) and R-groups of its first coordination sphere ligands His511, His385 and Cys334. In addition, the residue R-groups of His309, Asp383, Gln381, Arg363 and Lys465 were also included while the ThrAMP and tRNA^Thr^ substrates were modeled by methyl monophosphate threonyl and 1′,5′-deoxyribose, respectively [58]. The surrounding protein environment was modeled using a PCM method with a dielectric constant of 4.0. Relative energies were obtained at the IEF-PCM(*ɛ* = 4.0)-B3LYP/(6-311 + G(2df,p))//B3LYP/6-31G(d,p) level of theory.

**Figure 1.**
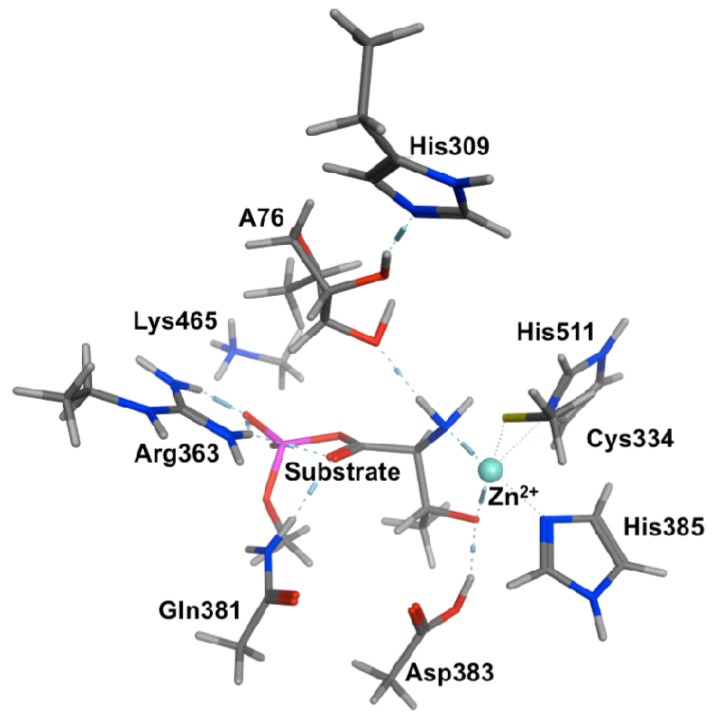
The Michaelis complex model (PRC) used for QM-cluster calculations in ThrRS including a neutral His309.

Within the X-ray crystal structures it was noted that the threonyl moiety bidentatively ligates to the Zn(II) ion via both its Thr-NH_2_ and R-group hydroxyl. This substrate-bound active site complex was indeed found to be the lowest in energy (Figure 1). However, facilitated by a hydrogen bond between the R-group hydroxyl and the carboxylate of the nearby Asp383 residue, the Zn(II)···(H_2_)N-Thr interaction was calculated to quite readily cleave to give an only very slightly higher energy reactive complex (RC). Notably, in the latter complex the Thr-NH_2_ group hydrogen bonds to the Ado76-3′-OH moiety.

We then systematically examined the proposed catalytic mechanism as well as possible alternatives. Specifically, we first considered mechanisms by which His309-H^+^ may act as a catalytic acid. The lowest energy pathway was found to involve proton transfer from His309-H^+^ onto the Ado76-2′-OH group. The latter concomitantly transferred its own proton via active site water onto the Thr-AMP’s carbonyl oxygen. The overall barrier for this mechanism was calculated to be quite high at approximately 141.4 kJ mol^−1^. Meanwhile, no mechanistic pathway could be identified at the above level of theory in which a neutral His309 acts as a base to abstract a proton from the Ado76-2′-OH group as experimentally proposed [63,64].

**Scheme 5.**
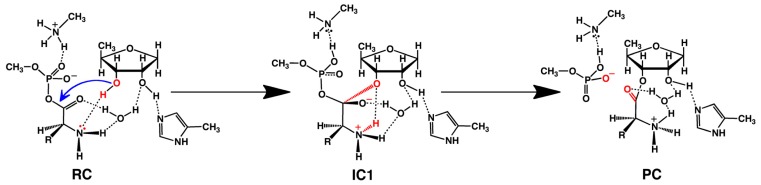
The overall computationally obtained mechanism for aminoacylation as catalyzed by threonyl-tRNA synthetase (see text).

However, as noted above, in the optimized structure of the RC complex involving a neutral His309, the substrate’s own Thr-NH_2_ group is hydrogen bonded to the Ado76-3′-OH moiety. That is, cleavage of the Zn(II)···(H_2_)N-Thr interaction allows the Thr-NH_2_ to re-position itself to accept the Ado76-3′-OH proton. Indeed, beginning from such an RC direct nucleophilic attack of the Ado76-3′-OH oxygen at the substrates carbonyl carbon centre occurs with a much lower barrier of only 105.1 kJ mol^−1^. Furthermore, this step proceeds with concomitant transfer of the Ado76-3′-OH proton onto the Thr-NH_2_ (*i.e.*, forms Thr-NH_3_^+^) as shown in Scheme 5. The resulting tetrahedral oxyanion intermediate (IC1) was calculated to lay only 52.8 kJ mol^−1^ higher in energy than the reactive complex RC. In the subsequent step, the C_substrate_–OP bond within the former aminoacyl-AMP moiety is cleaved with effectively no barrier. This gives the final product complex PC lying 15.8 kJ mol^−1^ lower in energy than RC, in which the threonyl moiety has now been transferred onto the Ado76-3′-oxygen.

Thus, computationally, it is suggested that ThrRS also uses a substrate-assisted catalytic mechanism. Now, however, due in part to the presence of the Zn(II) and lability of the Zn(II)···(H_2_)N-Thr interaction, the -NH_2_ moiety is instead able to act as the required mechanistic base.

### 2.3. Substrate Specificity and Initial Mechanisms of *S*-ribosylhomocysteinase (LuxS)

Conventionally, problems pertaining to enzymatic mechanisms can be placed into either one of two categories: specificity and rate-enhancement [66]. The former concerns itself with the ways in which substrates interact with enzymes’ binding site components, while the latter pertains to factors involved in the lowering of energy barriers including those that stabilize transition states and/or destabilize reactant/intermediates complexes. While these serve as separate conjectural models, they are in fact interdependent; insights into one can provide information on the other.

*S*-Ribosylhomocysteinase (LuxS) is a non-heme iron-containing enzyme that plays a key role in type-2 quorum sensing (QS-2), an important intra-organism communication and regulation process that helps regulate gene expression and microbial cell population [17]. QS-2 has been suggested to act as a universal language amongst bacterial species. Due to its possible involvement in the spread of infections, it has been suggested as a potential target for inhibition and LuxS inhibitors in particular could serve as broad-spectrum antibiotics [67].

**Scheme 6.**
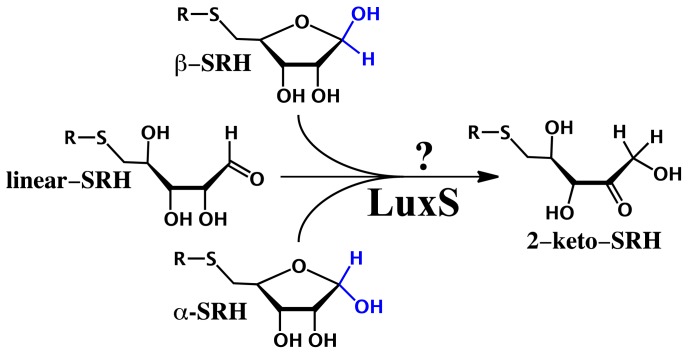
Schematic representation of the conjectured conversion of *S*-ribosylhomocysteine (α-SRH), β-SRH and linear-SRH into common putative 2-keto mechanistic intermediate.

From a chemical perspective, LuxS catalyzes the conversion of S-ribosylhomocysteine (SRH) into homocysteine and 4,5-dihydroxy-2,3-pentadione (DPD). Interestingly, it cleaves a stable thioether bond and mediates an internal redox reaction without the help of any redox-active cofactors. Despite the many experimental studies investigating its mechanism [67–70], many key fundamental questions remain including the nature of the substrate when bound in the active site. More specifically, SRH can co-exist in solution in three forms: two cyclic (α- and β-) furanose-containing SRH anomers and a corresponding linear aldose form (linear-SRH). Each one has quite distinct functionality and/or stereochemistry and thus, given that enzymes are often highly stereospecific it is unknown if LuxS binds two or more of these forms. Consequently, how the enzymes LuxS could perform its catalytic function on two or more of these substrates is unknown. For instance, could it possibly proceed via a common intermediate (Scheme 6)?

By a synergetic application of docking, MD and QM/MM for each of the possible substrate isomers we investigated the nature of the substrate-bound active site and the possible initial mechanistic reaction steps [17]. LuxS···substrate complex models were generated starting from the X-ray crystal structure of a C84A Co(II)-substituted LuxS with a presumed 2-keto-ribose intermediate (2-keto-SRH) (PDB:1YCL). Co(II) was replaced with Fe(II) and the mutation was reversed to regenerate the wild-type active site. A LuxS···linear-SRH model was modeled using the 2-keto-SRH as template while α-SRH and β-SRH were docked in the active site. The resulting models were sequentially minimized using the AMBER99 molecular mechanics force field. During MD simulations, annealing and production stages were performed in conjunction with the AMBER99 forcefield for an overall duration of 5 ns. During production stages a constant temperature of 300 K was used and residues within 13.5 Å of the substrate were free to move.

For both the α-SRH···LuxS and β-SRH···LuxS complexes the variation of the root-mean square deviations (RMSD) of the substrates’ atomic positions within the binding site in every snapshot during the course of the 5 ns simulation was analyzed, *i.e.*, their degree of flexibility and consistency in positioning. Average structures obtained from clusters analyses over the simulations clearly indicated that both cyclic anomers maintained consistent active site-substrate associations over the course of the simulations. Specifically, the O2 and O3 ribosyl oxygens remained ligated to the Fe(II) centre throughout the MD with average distances no greater than 2.30 Å. Simultaneously, the ribosyl –O3H hydroxyl group maintained a hydrogen bond with the anionic carboxylate of a nearby active site glutamate (E57) residue with average distances of 2.52 Å or less. Meanwhile, the cysteinyl (C84) that has been suggested to be catalytically essential was found to be positioned near both substrates ribosyl –C2H– moiety; *i.e.*, suitably positioned for proton abstraction as has been experimentally proposed [71]. The RMSD’s of the active site residues over the course of the 5 ns simulation were also considered and found to vary within a quite narrow range of approximately only 0.15–0.38 Å. That is, both cyclic possible substrates as well as the active site residues exhibit limited fluxional behavior with consistent positioning throughout the simulations.

In contrast, the bound linear-SRH conformation was more dynamic; cluster analysis of the MD simulation and the corresponding calculated RMSD’s indicated that it alternates between two distinct conformations. In the preferred conformation, with RMSD’s in the range of 0.35–0.55 Å, Cys84 was not positioned above the ribosyl C2H moiety. Furthermore, there are a reduced number of active site residue···linear-SRH interactions. Thus, while LuxS can bind all three SRH isoforms, the stability of the bound linear-SRH···LuxS complex is lower than that of both bound cyclic α-/β-SRH···LuxS complexes.

Possible initial steps for the catalyzed interconversion of the cyclic- and linear-SRH forms were then considered using an ONIOM(QM/MM)-based methodology [17]. We first considered a ribosyl ring opening mechanism starting from bound α- and β-SRH in which the C1–OH proton transfers directly to the ring oxygen O4 to form the respective bound linear-SRH complexes. While in both cases this was a concerted mechanism, *i.e.*, ring opening with proton transfer, the reaction barriers for α- and β-SRH ring opening processes were 207.6 and 155.6 kJ mol^−1^ respectively. Thus, such “direct” interconversion mechanisms are unlikely to be feasible.

**Figure 2.**
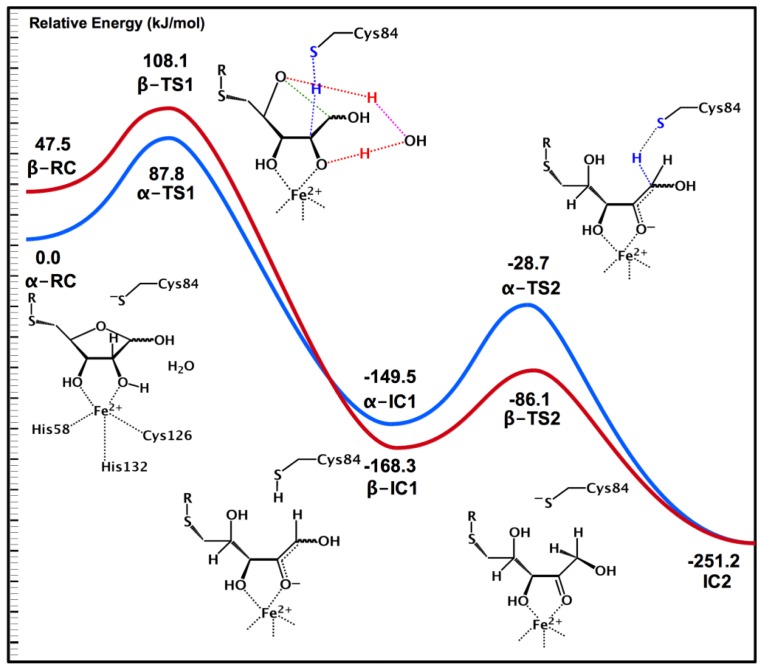
Potential energy profile for conversion of for α- and β-SRH into a 2-keto-SRH intermediate via a water-mediated ring opening process. The blue/red potential energy surface corresponds to the α/β-SRH conversion, respectively. The colored lines in the transition state schematic structures correspond to the particular bonds being broken and formed.

Experimentally, it had been suggested that LuxS may catalyze the ring opening [68]. Indeed, within all three MD obtained solvated structures of the bound active sites, a water molecule was ligated to the Fe(II) centre. For both α- and β-SRH, the Fe(II) acts as a Lewis acid, thereby increasing the acidity of coordinated ribosyl –O2H group. As a result, it is able to transfer its proton onto the active site water which concomitantly transfers a proton onto the ribosyl ring oxygen O4. Simultaneously for both anomers, the Cys84 thiolate deprotonates the ribosyl –C2H– moiety. Notably, for α- and β-SRH, this step occurs with barriers of 87.8 and 60.6 kJ mol^−1^ respectively (Figure 2); barriers that are enzymatically feasible. This step leads to the respective ring-opened intermediates α- and β-IC1 lying significantly lower in energy than their corresponding bound active site complexes α- and β-RC. Subsequently, for both α- and β-IC1, the now neutral thiol of Cys84 then transfers its –SH proton onto the intermediates C1 centre via α- and β-TS1 with barriers of 120.8 and 82.2 kJ mol^−1^, respectively (see Figure 1). It is noted that both of these transition structures are themselves markedly lower in energy than the lowest energy reactant complex α-RC. Remarkably, for both the α- and β-anomers this last step results in formation of a common intermediate, 2-keto-SRH that has been experimentally [72] shown to be a mechanistically viable intermediate.

In the case of linear-SRH, the Fe(II) again ligates to the substrate via the latter 2-OH and 3-OH oxygens. As a result of this interaction the –O2H is able to readily act as an acid, transferring its proton onto the nearby C1=O oxygen. Concomitantly, the R-group thiolate of Cys84 abstracts a proton from the ribosyl –C2H– moiety. This reaction occurs with a barrier of just 6.4 kJ mol^−1^ and gives the intermediate α-IC1. Importantly, the latter was also encountered in the water-assisted ring opening mechanism for active site bound β-SRH [17].

The coupling of docking, MD and QM/MM methods proved to particularly insightful when examining substrate binding by LuxS and the subsequent initial reaction steps. As seen above, they reveal that LuxS can bind all three possible substrates—α-SRH, β-SRH and linear-SRH—with varying stability. Subsequent elucidation of possible catalytic mechanisms via the use of QM/MM showed how active site water may facilitate opening of the ribosyl-ring in α-SRH and β-SRH. Importantly, it also showed how LuxS may bind its *S*-ribosylhomocysteine substrate in any of its forms and then convert all to a common mechanistic intermediate 2-keto-SRH (IC2) as illustrated in Figure 2.

### 2.4. MsrA and Its Reductive Ability: A Docking, MD and Multi-Model QM/MM Study

Methionine sulfoxide reductases (Msr’s) are a ubiquitous class of antioxidant enzymes that reduce methionine sulfoxide (MetSO) to methionine. Msr’s are commonly divided into two subclasses, labeled as A and B, that have quite distinct active sites. Notably, however, they are stereospecific for the S- and R-epimers of MetSO, respectively [73]. Due in part to their antioxidant roles in protein biochemistry, they are believed to play key roles in aging and age related diseases [74–76]. Indeed, for several species it has been shown that the overexpression of MsrA increases their life span [77,78].

Regardless of the class and their active site differences, Msr’s are generally believed to utilize a common mechanism [79]. More specifically, it is believed that the mechanism starts with the activation of an active site cysteinyl residue, known as the catalytic cysteinyl (Cys_cat_), by an unknown base followed by a subsequent nucleophilic attack of Cys_cat_ on the R-group sulfur center of methionine sulfoxide (MetSO). This results in the formation of a hypervalent sulfurane species in which Cys_cat_ is now covalently linked with the MetSO sulfur [80]. This occurs concomitantly with protonation of the R-group MetSO S=O oxygen by an active site acid. For MsrA mutation of an active site glutamyl (Glu) to alanyl drastically reduced the rate of catalysis, suggesting a possible role in activating Cys_cat_ [81]. However, X-ray crystal structures of MsrA generally show large distances between the R-groups Glu and Cys_cat_ [82]. In addition, single mutation of either of the two MsrA active site tyrosyls to phenylalanyl had insignificant effects on catalysis, while their double mutation had drastic effects [81]. Nevertheless, the sulfurane intermediate then undergoes further reaction to give methionine (Met) and a Cys_cat_ derived sulfenic acid (Cys_cat_SOH).

The mechanism by which such a species is formed is much debated in the literature [73,83]. This is partly due to the fact that sulfenic acid is a highly reactive species and thus, does not lend itself to experimental characterization or even identification [84]. This renders the application of computational methods particularly useful. It had previously been suggested that the Cys_cat_SOH sulfenic oxygen was obtained from the sulfoxide of MetSO. However, recent experimental studies have proposed that it is instead derived from the aqueous solvent [73]. Finally, a second active site cysteinyl, known as the recycling cysteine (Cys_Rec_), nucleophilically attacks the sulfur centre of Cys_cat_SOH to give an intramolecular disulfide bond (Cys_cat_S–SCys_Rec_) and a water molecule.

Through a complementary modeling approach, we recently investigated the catalytic mechanism of an MsrA from *Mycobacterium tuberculosis* [81]. An X-ray crystallographic structure of a complex of MsrA with a protein-bound methionine (PDB code: 1 NWA) was used as a template for further calculations [81]. In particular, due to a paucity of X-ray crystallographic information with regards to the actual enzyme-bound substrate MetSO, a thermally relaxed (at 298 K) solvated enzyme-substrate structure (*i.e.*, potential Michaelis complexes) was obtained by first docking MetSO within MsrA. Then, the 30 top-scoring structures were minimized using an MM method with the “best” then subjected to solvation and thermal relaxation via appropriate 1 ns MD simulations [81].

Importantly, analysis of the structures encountered over the course of the MD simulations clearly indicated a consistent hydrogen bond bridge via a water molecule between the thiol of the recycling cysteinyl (Cys13) and the carboxylate of an active site glutamyl (Glu52) which has been proposed to be important [82]. This suggests that Glu52 may act indirectly (*i.e.*, via a water) to activate the mechanistically key Cys13 residue. Furthermore, consistent strong hydrogen bonding interactions were observed between the substrate’s sulfoxide oxygen and the R-group phenols of two active site tyrosyl residues (Tyr44 and Tyr92). A suitable chemical model of the substrate-bound active site was derived from the average structure of the MD simulation [81] for use in the subsequent ONIOM (QM/MM) investigation on possible reaction steps leading to formation of an enzyme derived sulfenic acid (*i.e.*, Cys13S-OH). More specifically, optimized structures, vibrational frequencies and Gibb’s free energy corrections (Δ*G*_cor_) were obtained at the ONIOM (B3LYP/6-31G(d):AMBER96) level of theory within a mechanical embedding (ME) formalism. Relative Gibb’s free energies were subsequently determined via single point calculations on the above structures at the ONIOM (B3LYP/6-311 + G(2df,p):AMBER96) level of theory within an electrostatic embedding (EE) formalism with inclusion of the appropriate Δ*G*_cor_. The latter use of EE allows one to better model the effects of the non-homogeneous protein environment surrounding the active site region.

**Scheme 7.**
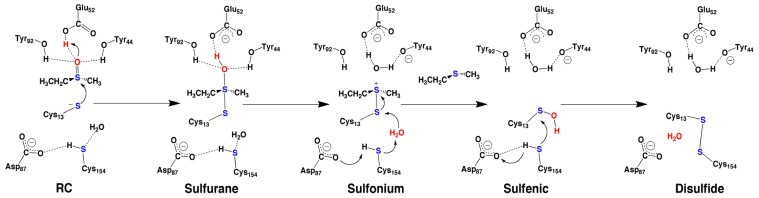
Schematic representation of the computationally proposed mechanism for the reduction of methionine sulfoxide via sulfenic acid to disulfide.

Notably, the QM/MM studies indicated that a substrate-bound pre-reactive complex (PRC) in which Cys13 was neutral (*i.e.*, Cys13SH) and Glu52 anionic (*i.e.*, Glu52COO^−^), could interconvert to a reactive complex (RC) in which Cys13 had been deprotonated (*i.e.*, Cys13S^−^) by Glu52 at a free energy cost of 49.3 kJ mol^−1^ (Scheme 7). In the next step, the sulfur of Cys13S^−^ nucleophilically attacks the sulfur of the MetSO substrate, with concomitant proton transfer from the now neutral Glu52COOH group onto the MetSO sulfoxide oxygen with a small barrier of only 11.8 kJ mol^−1^. The resulting sulfurane intermediate is predicted to lay almost thermoneutral with PRC, being 47.0 kJ mol^−1^ lower in free energy than RC. As noted above, mutation of both Tyr44 and Tyr92 to Phe significantly inhibited catalysis, in contrast to that observed for single mutation of either Tyr [82]. Notably, the sulfurane oxygen remains hydrogen bonded to both R-group –OH of Tyr44 and Tyr92. Indeed, either Tyr-OH group is then able to act as an acid. Specifically, they protonate the sulfurane oxygen, with essentially no reaction barrier, to give H_2_O and a sulfonium intermediate lying a further 7.0 or 40.4 kJ mol^−1^ lower in energy depending on whether the proton was donated by Tyr92 or Tyr44, respectively. The thiol of the recycling cysteinyl (Cys154) is hydrogen bonded to a nearby carboxyl of Asp87. Furthermore, a water molecule was observed in the MD simulations and in the ONIOM studies to be consistently positioned near the Cys13 sulfur, and hydrogen bonded with the thiol of Cys154. Consequently, Cys154 is able to help activate the H_2_O for nucleophilic attack at the Cys13 sulfur of the sulfonium intermediate. This leads to formation of free Met, *i.e.*, reduced MetSO, and a Cys13-derived sulfenic acid lying markedly lower in energy. That is, sulfenic acid formation is predicted to be a quite exergonic process. We also re-examined this process in which either Tyr92 or Tyr44 were mutated to a Phe residue. The reaction barriers did not change significantly, in agreement with experimental observations.

In many computational studies, MD simulations are used solely to generate a suitable structure of the bound-substrate active site. However, the free Met is now able to leave the active site and this may lead to changes in the active site. Hence, we performed an MD simulation on the sulfenic acid intermediate as summarized above, and obtained a suitable chemical model of the sulfenic acid intermediate for investigation of its subsequent reduction using the above ONIOM-based approach. Furthermore, the new-modeled QM layer is now different from the previous one as we focused on including the surrounding residues of Cys154 to better elucidate the mechanism. The reaction of Cys13SOH to give an intramolecular Cys13S–SCys154 disulfide bond was determined to be able to occur via a multistep process. More specifically, Asp87 indirectly acts via bridging water to deprotonate the thiol of Cys145 (*i.e.*, the recycling cysteinyl). The resulting complex, containing a now “activated” Cys154S^−^ moiety, lies only 51.8 kJ mol^−1^ higher in energy than the lowest energy Cys13SOH-containing intermediate. The Cys154S^−^ sulfur is then able to readily react with the Cys13SOH moiety with a barrier of only 39.4 kJ mol^−1^ to form the disulfide product (Figure 2). The reduction of the sulfenic acid is calculated to be a highly exergonic process with the disulfide product lying significantly lower in energy than the sulfenic acid intermediate complex [81].

Thus, the complementary use of docking, molecular dynamics and ONIOM (QM/MM) computational chemistry methods provided several key insights into the overall mechanism of MsrA (Scheme 7), including the role of key active site residues such as Glu52, Tyr92 and Tyr44. In addition, it also suggested possible mechanistic roles for other active site residues such as Asp87. Furthermore, our results complement previous experimental mutagenesis studies.

## 3. Conclusions

This review presented a variety of examples pertaining to the use of distinguished computational methods in deciphering enzyme catalysis. As shown herein, DFT clusters, QM/MM, molecular docking and MD simulations can be used, either in their own right or as members of an assembly in the investigation of enzymatic processes. As seen, in the absence of a crystal structure and while the nature of the biocatalytic environment has not been experimentally resolved, QM-DFT clusters can model and compare an extensive set of possible reactive species. In the cases where crystal structures exist but essential details pertaining to the active site’s protonation state and substrate’s binding conformation remain elusive, docking, MD simulations and QM/MM can be combined in the framework of a single analysis. In the cases where an enzymatic process involves the exit of a substrate during catalysis, MD simulations can model the resulting conformational changes while the use of different QM/MM models can model the catalysis. The synergistic use of experimental chemical methods coupled with divergent multi-scale methods, ranging from the quantum- to the statistical-mechanical, holds promise in uncovering the key to enzymatic catalysis.

## References

[1] Stodulski M., Gulder T. (2012). Nanoparticles and peptides: A fruitful liaison for biomimetic catalysis. Angew. Chem. Int. Ed.

[2] Lehn J.M., Benyus J., Swiegers G. (2012). Bioinspiration and Biomimicry in Chemistry: Reverse-Engineering Nature.

[3] Frushicheva M.P., Cao J., Chu Z.T., Warshel A. (2010). Exploring challenges in rational enzyme design by simulating the catalysis in artificial kemp eliminase. Proc. Natl. Acad. Sci. USA.

[4] Llano J., Gauld J.W. (2010). Mechanistics of Enzyme Catalysis: From Small to Large Active-Site Models. Quantum Biochemistry.

[5] Garcia-Viloca M., Gao J., Karplus M., Truhlar D.G. (2004). How enzymes work: Analysis by modern rate theory and computer simulations. Science.

[6] Kamerlin S.C.L., Warshel A. (2010). At the dawn of the 21st century: Is dynamics the missing link for understanding enzyme catalysis?. Proteins: Struct. Funct. Bioinforma.

[7] Glowacki D.R., Harvey J.N., Mulholland A.J. (2012). Taking Ockham’s razor to enzyme dynamics and catalysis. Nat. Chem.

[8] Hammes G.G. (2002). Multiple conformational changes in enzyme catalysis. Biochemistry.

[9] Benkovic S.J., Hammes-Schiffer S. (2003). A perspective on enzyme catalysis. Science.

[10] Henzler-Wildman K.A., Lei M., Thai V., Kerns S.J., Karplus M., Kern D. (2007). A hierarchy of timescales in protein dynamics is linked to enzyme catalysis. Nature.

[11] Borowski T., Bassan A., Siegbahn P.E.M. (2004). Mechanism of dioxygen activation in 2-oxoglutarate-dependent enzymes: A hybrid DFT study. Chem. Eur. J.

[12] De Visser S.P., Kumar D., Cohen S., Shacham R., Shaik S. (2004). A predictive pattern of computed barriers for C–H hydroxylation by compound I of cytochrome P450. J. Am. Chem. Soc.

[13] Bushnell E.A.C., Gherib R., Gauld J.W. (2013). Insights into the catalytic mechanism of coral allene oxide synthase: A dispersion corrected density functional theory study. J. Phys. Chem. B.

[14] Van Gunsteren W.F., Luque F., Timms D., Torda A. (1994). Molecular mechanics in biology: From structure to function, taking account of solvation. Annu. Rev. Biophys. Biomol. Struct.

[15] Krüger P., Straßburger W., Wollmer A., van Gunsteren W.F. (1985). A comparison of the structure and dynamics of avian pancreatic polypeptide hormone in solution and in the crystal. Eur. Biophys. J.

[16] Levy Y., Onuchic J.N. (2006). Water mediation in protein folding and molecular recognition. Annu. Rev. Biophys. Biomol. Struct.

[17] Huang W., Gherib R., Gauld J.W. (2012). An active site water broadens substrate specificity in *S*-ribosylhomocysteinase (LuxS): A docking, MD, and QM/MM study. J. Phys. Chem. B.

[18] Barillari C., Taylor J., Viner R., Essex J.W. (2007). Classification of water molecules in protein binding sites. J. Am. Chem. Soc.

[19] Storm D.R., Koshland D.E. (1970). A source for the special catalytic power of enzymes: Orbital steering. Proc. Natl. Acad. Sci. USA.

[20] Jonsson T., Glickman M.H., Sun S., Klinman J.P. (1996). Experimental evidence for extensive tunneling of hydrogen in the lipoxygenase reaction: Implications for enzyme catalysis. J. Am. Chem. Soc.

[21] Karplus M., Kuriyan J. (2005). Molecular dynamics and protein function. Proc. Natl. Acad. Sci. USA.

[22] Warren G.L., Andrews C.W., Capelli A.M., Clarke B., Lalonde J., Lambert M.H., Lindvall M., Nevins N., Semus S.F., Senger S. (2006). A critical assessment of docking programs and scoring functions. J. Med. Chem.

[23] Karplus M., McCammon J.A. (2002). Molecular dynamics simulations of biomolecules. Nat. Struct. Mol. Biol.

[24] Siegbahn P.E.M., Himo F. (2011). The quantum chemical cluster approach for modeling enzyme reactions. Wiley Interdiscip. Rev. Comput. Mol. Sci.

[25] Himo F. (2006). Quantum chemical modeling of enzyme active sites and reaction mechanisms. Theor. Chem. Acc.

[26] Siegbahn P.E.M., Blomberg M.R.A. (2000). Transition-metal systems in biochemistry studied by high-accuracy quantum chemical methods. Chem. Rev.

[27] Siegbahn P.E.M., Borowski T. (2006). Modeling enzymatic reactions involving transition metals. Acc. Chem. Res.

[28] Becke A.D. (1993). Density-functional thermochemistry. III. The role of exact exchange. J. Chem. Phys.

[29] Cornell W.D., Cieplak P., Bayly C.I., Gould I.R., Merz K.M., Ferguson D.M., Spellmeyer D.C., Fox T., Caldwell J.W., Kollman P.A. (1995). A second generation force field for the simulation of proteins, nucleic acids, and organic molecules. J. Am. Chem. Soc.

[30] Wang J., Wolf R.M., Caldwell J.W., Kollman P.A., Case D.A. (2004). Development and testing of a general amber force field. J. Comput. Chem.

[31] Pérez A., Marchán I., Svozil D., Sponer J., Cheatham T.E., Laughton C.A., Orozco M. (2007). Refinement of the AMBER force field for nucleic acids: Improving the description of α/γ conformers. Biophys. J.

[32] Meagher K.L., Redman L.T., Carlson H.A. (2003). Development of polyphosphate parameters for use with the AMBER force field. J. Comput. Chem.

[33] MacKerell A.D., Banavali N., Foloppe N. (2000). Development and current status of the CHARMM force field for nucleic acids. Biopolymers.

[34] Vanommeslaeghe K., Hatcher E., Acharya C., Kundu S., Zhong S., Shim J., Darian E., Guvench O., Lopes P., Vorobyov I. (2010). CHARMM general force field: A force field for drug-like molecules compatible with the CHARMM all-atom additive biological force fields. J. Comput. Chem.

[35] Schmidt J.M., Brueschweiler R., Ernst R.R., Dunbrack R.L., Joseph D., Karplus M. (1993). Molecular dynamics simulation of the proline conformational equilibrium and dynamics in antamanide using the CHARMM force field. J. Am. Chem. Soc.

[36] Van Gunsteren W.F., Daura X., Mark A.E. (2002). GROMOS Force Field. Encyclopedia of Computational Chemistry.

[37] Oostenbrink C., Soares T., Vegt N.A., van Gunsteren W.F. (2005). Validation of the 53A6 GROMOS force field. Eur. Biophys. J.

[38] Schmid N., Eichenberger A., Choutko A., Riniker S., Winger M., Mark A.E., van Gunsteren W.F. (2011). Definition and testing of the GROMOS force-field versions 54A7 and 54B7. Eur. Biophys. J.

[39] Kaminski G.A., Friesner R.A., Tirado-Rives J., Jorgensen W.L. (2001). Evaluation and reparametrization of the OPLS-AA force field for proteins via comparison with accurate quantum chemical calculations on peptides. J. Phys. Chem. B.

[40] Kahn K., Bruice T.C. (2002). Parameterization of OPLS–AA force field for the conformational analysis of macrocyclic polyketides. J. Comput. Chem.

[41] Kony D., Damm W., Stoll S., van Gunsteren W.F. (2002). An improved OPLS–AA force field for carbohydrates. J. Comput. Chem.

[42] Warshel A., Levitt M. (1976). Theoretical studies of enzymic reactions: Dielectric, electrostatic and steric stabilization of the carbonium ion in the reaction of lysozyme. J. Mol. Biol.

[43] Lin H., Truhlar D.G. (2007). QM/MM: What have we learned, where are we, and where do we go from here?. Theor. Chem. Acc.

[44] Senn H.M., Thiel W. (2007). QM/MM studies of enzymes. Curr. Opin. Chem. Biol.

[45] Chung L.W., Hirao H., Li X., Morokuma K. (2012). The ONIOM method: Its foundation and applications to metalloenzymes and photobiology. Wiley Interdiscip. Rev. Comput. Mol. Sci.

[46] Plewczynski D., Łaźniewski M., Augustyniak R., Ginalski K. (2011). Can we trust docking results? Evaluation of seven commonly used programs on PDBbind database. J. Comput. Chem.

[47] Durrant J., McCammon J.A. (2011). Molecular dynamics simulations and drug discovery. BMC Biol.

[48] Ziegler T., Autschbach J. (2005). Theoretical methods of potential use for studies of inorganic reaction mechanisms. Chem. Rev.

[49] Van der Kamp M.W., Mulholland A.J. (2013). Combined quantum mechanics/molecular mechanics (QM/MM) methods in computational enzymology. Biochemistry.

[50] Nobelprize.org http://www.nobelprize.org/nobel_prizes/chemistry/laureates/2013/.

[51] Vogt R.N., Spies H.S.C., Steenkamp D.J. (2001). The biosynthesis of ovothiol A (*N*^1^-methyl-4-mercaptohistidine). Eur. J. Biochem.

[52] Braunshausen A., Seebeck F.P. (2011). Identification and characterization of the first ovothiol biosynthetic enzyme. J. Am. Chem. Soc.

[53] Bushnell E.A.C., Fortowsky G.B., Gauld J.W. (2012). Model iron, oxo species and the oxidation of imidazole: Insights into the mechanism of OvoA and EgtB?. Inorg. Chem.

[54] Cances E., Mennucci B., Tomasi J. (1997). A new integral equation formalism for the polarizable continuum model: Theoretical background and applications to isotropic and anisotropic dielectrics. J. Chem. Phys.

[55] De Visser S.P., Straganz G.D. (2009). Why do cysteine dioxygenase enzymes contain a 3-his ligand motif rather than a 2his/1Asp motif like most nonheme dioxygenases?. J. Phys. Chem. A.

[56] Fredrick K., Ibba M. (2009). Protein synthesis errors rectified in retrospect. Nature.

[57] Gromadski K.B., Rodnina M.V. (2004). Kinetic determinants of high-fidelity tRNA discrimination on the ribosome. Mol. Cell.

[58] Guth E.C., Francklyn C.S. (2007). Kinetic discrimination of tRNA identity by the conserved motif 2 loop of a class II aminoacyl-tRNA synthetase. Mol. Cell.

[59] Huang W., Bushnell E.A.C., Francklyn C.S., Gauld J.W. (2011). The α-amino group of the threonine substrate as the general base during tRNA aminoacylation: A new version of substrate-assisted catalysis predicted by hybrid DFT. J. Phys. Chem. A.

[60] Guth E., Connolly S.H., Bovee M., Francklyn C.S. (2005). A substrate-assisted concerted mechanism for aminoacylation by a class II aminoacyl-tRNA synthetase. Biochemistry.

[61] Liu H., Gauld J.W. (2008). Substrate-assisted catalysis in the aminoacyl transfer mechanism of histidyl-tRNA synthetase: A density functional theory study. J. Phys. Chem. B.

[62] Bushnell E.A.C., Huang W., Llano J., Gauld J.W. (2012). Molecular dynamics investigation into substrate binding and identity of the catalytic base in the mechanism of threonyl-tRNA synthetase. J. Phys. Chem. B.

[63] Minajigi A., Francklyn C.S. (2008). RNA-assisted catalysis in a protein enzyme: The 2′-hydroxyl of tRNA (Thr) A76 promotes aminoacylation by threonyl-tRNA synthetase. Proc. Natl. Acad. Sci. USA.

[64] Francklyn C.S. (2008). DNA polymerases and aminoacyl-tRNA synthetases: Shared mechanisms for ensuring the fidelity of gene expression. Biochemistry.

[65] Sethi A., Eargle J., Black A.A., Luthey-Schulten Z. (2009). Dynamical networks in tRNA: Protein complexes. Proc. Natl. Acad. Sci. USA.

[66] Jencks W.P. (1987). Catalysis in Chemistry and Enzymology.

[67] Pei D., Zhu J. (2004). Mechanism of action of *S*-ribosylhomocysteinase (LuxS). Curr. Opin. Chem. Biol.

[68] Gopishetty B., Zhu J., Rajan R., Sobczak A.J., Wnuk S.F., Bell C.E., Pei D. (2009). Probing the catalytic mechanism of *S*-ribosylhomocysteinase (LuxS) with catalytic intermediates and substrate analogues. J. Am. Chem. Soc.

[69] Rajan R., Zhu J., Hu X., Pei D., Bell C.E. (2005). Crystal structure of *S*-ribosylhomocysteinase (LuxS) in complex with a catalytic 2-ketone intermediate. Biochemistry.

[70] Zhu J., Dizin E., Hu X., Wavreille A.S., Park J., Pei D. (2003). *S*-Ribosylhomocysteinase (LuxS) is a mononuclear iron protein. Biochemistry.

[71] Schauder S., Shokat K., Surette M.G., Bassler B.L. (2001). The LuxS family of bacterial autoinducers: Biosynthesis of a novel quorum-sensing signal molecule. Mol. Microbiol.

[72] Zhu J., Hu X., Dizin E., Pei D. (2003). Catalytic mechanism of *S*-ribosylhomocysteinase (LuxS): Direct observation of ketone intermediates by ^13^C NMR spectroscopy. J. Am. Chem. Soc.

[73] Lim J.C., You Z., Kim G., Levine R.L. (2011). Methionine sulfoxide reductase A is a stereospecific methionine oxidase. Proc. Natl. Acad. Sci. USA.

[74] Ezraty B., Aussel L., Barras F. (2005). Methionine sulfoxide reductases in prokaryotes. BBA Proteins Proteomics.

[75] Weissbach H., Resnick L., Brot N. (2005). Methionine sulfoxide reductases: History and cellular role in protecting against oxidative damage. BBA Proteins Proteomics.

[76] Moskovitz J. (2005). Methionine sulfoxide reductases: Ubiquitous enzymes involved in antioxidant defense, protein regulation, and prevention of aging-associated diseases. BBA Proteins Proteomics.

[77] Ruan H., Tang X.D., Chen M.L., Joiner M.A., Sun G., Brot N., Weissbach H., Heinemann S.H., Iverson L., Wu C.F. (2002). High-quality life extension by the enzyme peptide methionine sulfoxide reductase. Proc. Natl. Acad. Sci. USA.

[78] Moskovitz J., Bar-Noy S., Williams W.M., Requena J., Berlett B.S., Stadtman E.R. (2001). Methionine sulfoxide reductase (MsrA) is a regulator of antioxidant defense and lifespan in mammals. Proc. Natl. Acad. Sci. USA.

[79] Boschi-Muller S., Olry A., Antoine M., Branlant G. (2005). The enzymology and biochemistry of methionine sulfoxide reductases. BBA Proteins Proteomics.

[80] Dokainish H.M., Gauld J.W. (2013). A molecular dynamics and quantum mechanics/molecular mechanics study of the catalytic reductase mechanism of methionine sulfoxide reductase A: Formation and reduction of a sulfenic acid. Biochemistry.

[81] Antoine M., Gand A., Boschi-Muller S., Branlant G. (2006). Characterization of the amino acids from *Neisseria meningitidis* MsrA involved in the chemical catalysis of the methionine sulfoxide reduction step. J. Biol. Chem.

[82] Taylor A.B., Benglis D.M., Dhandayuthapani S., Hart P.J. (2003). Structure of *Mycobacterium tuberculosis* methionine sulfoxide reductase A in complex with protein-bound methionine. J. Bacteriol.

[83] Thiriot E., Monard G., Boschi-Muller S., Branlant G., Ruiz-López M.F. (2011). Reduction mechanism in class A methionine sulfoxide reductases: A theoretical chemistry investigation. Theor. Chem. Acc.

[84] Poole L.B., Karplus P.A., Claiborne A. (2004). Protein sulfenic acids in redox signaling. Annu. Rev. Pharmacol. Toxicol.

